# Teaching and learning the Hodgkin-Huxley model based on software developed in NEURON’s programming language *hoc*

**DOI:** 10.1186/1472-6920-13-70

**Published:** 2013-05-15

**Authors:** Oscar E Hernández, Eduardo E Zurek

**Affiliations:** 1 Departamento de Química y Biología, Universidad del Norte, Barranquilla, Atlántico, Colombia; 2 Departamento de Ingeniería de Sistemas, Universidad del Norte, Barranquilla, Atlántico, Colombia

## Abstract

**Background:**

We present a software tool called SENB, which allows the geometric and biophysical neuronal properties in a simple computational model of a Hodgkin-Huxley (HH) axon to be changed. The aim of this work is to develop a didactic and easy-to-use computational tool in the NEURON simulation environment, which allows graphical visualization of both the passive and active conduction parameters and the geometric characteristics of a cylindrical axon with HH properties.

**Results:**

The SENB software offers several advantages for teaching and learning electrophysiology. First, SENB offers ease and flexibility in determining the number of stimuli. Second, SENB allows immediate and simultaneous visualization, in the same window and time frame, of the evolution of the electrophysiological variables. Third, SENB calculates parameters such as time and space constants, stimuli frequency, cellular area and volume, sodium and potassium equilibrium potentials, and propagation velocity of the action potentials. Furthermore, it allows the user to see all this information immediately in the main window. Finally, with just one click SENB can save an image of the main window as evidence.

**Conclusions:**

The SENB software is didactic and versatile, and can be used to improve and facilitate the teaching and learning of the underlying mechanisms in the electrical activity of an axon using the biophysical properties of the squid giant axon.

## Background

Computational Neuroscience is a field of knowledge that creates models of individual neurons and biological neural networks of any part of the nervous system. In addition to supporting scientific research, Computational Neuroscience can be used to create computational models for teaching neuroscience, and thus for teaching electrophysiology [1-5]. Different strategies can be applied in the teaching and learning of the basic concepts of neuronal electrophysiology, including reading text-books and journal papers, experimental observation, and computational simulations.

### Reading text-books and journal papers

Students are guided through readings and static figures found in text-books and journal papers, and also figures predesigned by the instructor, with the aim of understanding the behavior of different variables. However, this strategy does not allow any opportunities for interactively exploring new results arising from variations in the different neuronal conduction parameters [6,7].

### Experimental observation

Students carry out experiments to observe the temporal or spatial evolution of the variables. This strategy facilitates the understanding of concepts related to the properties of a specific neuron or neuronal circuit, but it depends on the conditions under which the experiments were performed [8,9]. In addition, some neuronal electrophysiological phenomena are difficult to verify experimentally.

### Computer simulations

Electrophysiological phenomena can be simulated through the use of software [1-5]. This offers multiple alternatives for modifying the electrical neuronal conduction properties, environmental conditions, and neuronal geometry, and for calculating and visualizing graphically the temporal or spatial evolution of the studied variables. Thus, computational simulation is an excellent option to overcome the difficulties present in the strategies mentioned above.

The need to use didactic software became evident during the development of a course of Neuronal Electrophysiology for undergraduate students in the Medicine program at Universidad del Norte (Barranquilla, Colombia). Such software must allow for the modification of the geometrical properties of a cylindrical axon, such as its length (L) and diameter (diam). The software must also permit the modification of neuronal biophysical properties such as the properties of a squid giant axon [10]; in this work, these are called “HH” properties.

Currently, there are several specialized software packages available for visualizing neural phenomena from different perspectives. These include NEST, which uses unicompartmental models [11], and NEURON and GENESIS, which use both uni- and multi-compartmental models, thereby providing a more realistic model [12-15]. Of these packages, NEURON is the most popular, with numerous papers published in prestigious journals in the neuroscience field [16,17]. The literature clearly shows its efficiency in developing neuronal simulations with full control of the morphological and biophysical properties.

On the other hand, it is important to highlight that, by default, NEURON uses the kinetics of the potassium and sodium channels with HH properties. Furthermore, to enable the opening and closing rates of the channels to adapt to changes in temperature [18], NEURON uses a temperature coefficient (k) defined as

(1) $$k=Q_{10}^{\frac{T-T_{o}}{10}}$$

where *Q*_10_ is a measure of the increase in the opening and closing rates of the HH ion channels when the temperature (T) rises 10°C above the laboratory temperature, *T*_0_=6.3°C (internal temperature of the squid giant axon) [19].

The SENB software enables simulation of the propagation of action potential through a Hodgkin-Huxley type axon. SENB also allows the user to appreciate the effect of changing the geometric parameters (L and diam) of the axon, current stimuli parameters (NoStim, Amp, Dur, and Delay), active (GNa and GK) and passive (GL, Ra, and Cm) conduction parameters, temperature (T) of the axon environment, and internal and external ionic concentrations. The effects of changing these parameters can be observed in the temporal evolution of the membrane current and potential, the leak current, the sodium and potassium current, and the gating variables, as well as in the membrane resistance, the time and space constants, the sodium, potassium, and leak equilibrium potential, and the propagation speed of the action potential. The variables related to the parameters described above are explained in the Implementation section.

Users with programming experience can develop software using a combination of *hoc* (an interpreter with C-like syntax [20]) and Python [21], and can add new membrane properties with the compiled NMODL language [22]. For examples of these, refer to the web page in reference [23]. This software is aimed at special cases and for use by expert users, and thus does not facilitate teaching and learning processes. Note that there is a commercially available didactic software package, called “NEURON in action” [4], which is also based on the NEURON environment.

## Implementation

SENB has been designed as a numerical simulation in the NEURON simulation environment. The SENB software implementation facilitates changing both the passive and active conduction of the following: maximum specific sodium (GNa) and potassium (GK) conduction, specific leak conductance (GL), axoplasmic resistance (Ra), and specific membrane capacitance (Cm). Furthermore, changes in the geometric (L and diam) parameters of a cylindrical unmyelinated axon model with HH properties can easily be made. Details of the software are given below and the software can be downloaded from the URL given in [24].

## Details of the tool

SENB was developed using *hoc*, which is an interpreted programming language used to write scripts in NEURON. The software is composed of the following seven functional unit blocks: 

*Block 1*: Builds the geometry of a cylindrical axon so that the geometric parameters, such as L and diam, can be set.

**Figure 1:**
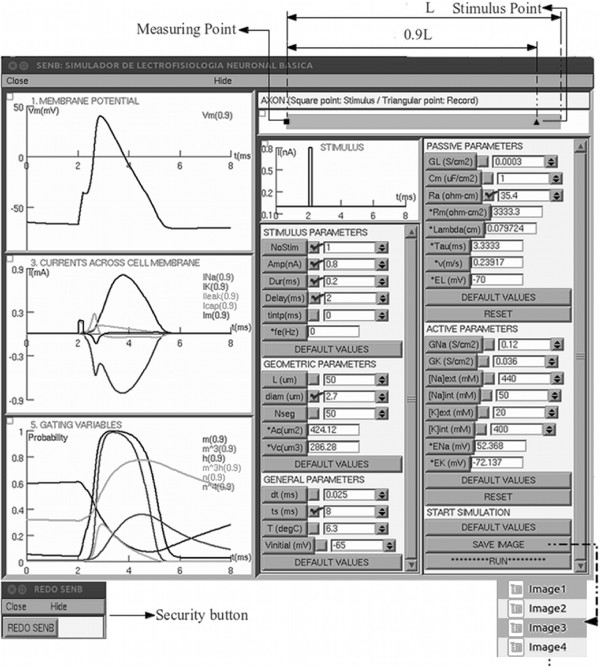
**Main Window of SENB.** The left side of the main window shows three graphs of the temporal evolution of the membrane potential, currents across the cell membrane, and gating variables, respectively. On the right side of the main window there are five data panels representing the passive, active, geometric, general, and stimulus parameters, as well as a panel with options to start the simulation. Note the graphs of the measuring point and stimulus point on the axon model at the top. The Security button at the bottom left and the Save image at the bottom right of the main window can be used to provide evidence. Furthermore, the values for fe, Lambda, Tau, v, EL, ENa, EK, Ac, and Vc are calculated automatically.

*Block 2*: Allows for the placement, in the axon model, of the active (HH properties) and passive (cable properties) parameters. This includes a numerical calculation of the sodium (ENa, Eq. 6) and potassium (EK, Eq. 7) equilibrium potentials using a computational implementation. Leak equilibrium potential (EL) is calculated by default in NEURON. The locations of the stimulus and recording electrodes cannot be changed, as shown in the top right of Figure 1.

*Block 3*: Simulates the stimulation electrode (path electrode in current clamp mode) and generates a graph of the current stimulus.

*Block 4*: Determines the default values for all parameters of the simulation, including time step (dt), total time (ts), T in °C, and the initial value of Vm (Vinitial).

*Block 5*: Generates graphs of the temporal evolution of the membrane potential, current densities, and gating variables. This block also generates an image of the axon model.

*Block 6*: Includes the numerical calculation of specific membrane resistance (Rm, Eq. 2), propagation velocity of the action potentials (v, Eq. 5), time constant (Tau, Eq. 3), space constant (Lambda, Eq. 4), and frequency of stimuli (fe, Eq. 8) using a computational implementation. It also contains a procedure for storing the images of the principal window as evidence.

*Block 7*: Designs the graphical user interface (GUI) of the main window as well as the security button called “REDO SENB” (depicted in the lower left of Figure 1), which allows the main window to be recovered in the event that it is accidentally closed. Furthermore, this block includes a numerical calculation of the area of the axonal membrane (Ac, Eq. 9) and axonal volume (Vc, Eq. 10).

(2) $$\begin{array}{ll} \;\text{Rm} & =1/\text{GL}\;\left(\Omega {\text{cm}}^{2}\right)\; \end{array}$$

(3) $$\begin{array}{ll} \;\text{Tau} & =10^{-3}\text{Cm}/\text{GL}\;(\text{ms})\; \end{array}$$

(4) $$\begin{array}{ll} \;\text{Lambda} & =10^{-2}\sqrt{d/4\text{Ra}\;\text{GL}}(\text{cm})\; \end{array}$$

(5) $$\begin{array}{ll} \;v & =10\frac{\text{Lambda}}{\text{Tau}}(m/s)\; \end{array}$$

(6) $$\begin{array}{ll} \;\text{ENa} & =\frac{1000(8.314)(273.15+T)}{96485.3}\text{log}\frac{[{\text{Na}}^{+}]\text{ext}}{[{\text{Na}}^{+}]\text{int}}\;(\text{mV})\; \end{array}$$

(7) $$\begin{array}{ll} \;\text{EK} & =\frac{1000(8.314)(273.15+T)}{96485.3}\text{log}\frac{[K^{+}]\text{ext}}{[K^{+}]\text{int}}\;(\text{mV})\; \end{array}$$

(8) $$\begin{array}{ll} \text{fe} & =10^{3}\frac{\text{NoStim}}{\mathrm{NoStim}(\text{dur})+(\text{NoStim}-1)(\text{tintp})}\;(\text{Hz})\; \end{array}$$

(9) $$\begin{array}{ll} \text{Ac} & =\pi \text{diamL}\left(\mu m^{2}\right)\; \end{array}$$

(10) $$\begin{array}{ll} \text{Vc} & =\frac{\pi {\text{diam}}^{2}L}{4}\left(\mu m^{3}\right)\; \end{array}$$

In the above equations, [Na]int and [Na]ext are the intra- and extracellular sodium concentrations, respectively, and [K]int and [K]ext are the intra- and extracellular potassium concentrations, respectively. NoStim is the number of square pulses or current stimuli and tintp is the time interval between these.

The sections of SENB’s graphical interface shown in Figure 1 are described below.

The graphs on the left side of the main window show the temporal evolution of certain variables. The upper graph, labeled “MEMBRANE POTENTIAL”, shows the membrane potential, Vm, while the graph in the center, labeled “CURRENTS ACROSS CELL MEMBRANE”, shows the sodium (INa), potassium (IK), leak (Ileak), capacitive, and membrane (Im) current densities. The lower graph, labeled “GATING VARIABLES”, depicts the probabilities of a voltage-sensor sodium channel being in an active state (m), a voltage-sensor potassium channel being in an active state (n), and the sodium channel inactivation gate being in a non-inactivated state (h), as well as the probabilities that the three voltage sensors in the sodium channel are in an active state (m^3^), and the sodium and potassium channels are open (m^3^h and n^4^).

On the right side of the main window, there are six data panels labeled “PASSIVE PARAMETERS”, “ACTIVE PARAMETERS”, “GEOMETRIC PARAMETERS”, “GENERAL PARAMETERS”, “STIMULUS PARAMETERS”, and “START SIMULATION”. Each of these is described below. 

 PASSIVE PARAMETERS: This panel allows the user to set the values of GL, Cm, and Ra and also shows the calculated values of Rm, Lambda, Tau, and EL. Furthermore, it has two buttons; one to assign a set of default values to the passive parameters and the other to reset the values (Reset).

 ACTIVE PARAMETERS: The values of GNa, GK, [Na]ext, [K]ext, [Na]int, and [K]int can be set in this panel, which also shows the calculated values of ENa and EK. The panel also has two buttons, “Reset” and “Default values”, which are similar to those in the Passive Parameter panel.

 GEOMETRIC PARAMETERS: This panel allows the geometrical parameters, L and diam, of an axon with a shape defined by a right circular cylinder (Figure 1) to be set. There is also a box that allows the assignation of a discretization parameter called “Nseg”, for greater accuracy in the calculation. Higher values should be assigned in this box (e.g., 2,..,100,..,400,...), although higher accuracy increases the simulation time. The calculated values of Ac and Vc are also shown in this panel.

 GENERAL PARAMETERS: The following parameters can be set: time step (dt), simulation time or tstop (ts), T in °C, and the initial value of Vm (Vinitial).

 STIMULUS PARAMETERS: Here the user can set the characteristics of the current stimuli (current clamp). This panel has been designed to facilitate the changing of the following parameters: NoStim, amplitude (Amp), and duration (Dur) of each current pulse, the time instant at which the current stimuli is initiated (Delay), and the time between square pulses of the current stimuli (tintp), which together with Dur determine fe (Eq. 8).

 START SIMULATION: This panel contains the buttons “SAVE IMAGE”, “RUN”, and “DEFAULT VALUES”. The “SAVE IMAGE” button allows for screen capture of the main window as evidence, assigning sequential names to each capture as it is generated. The “RUN” button initiates the simulation, while the “DEFAULT VALUES” button assigns a set of default values for all sections in the main window.

## Results and discussion

### Tool performance

**Figure 2:**
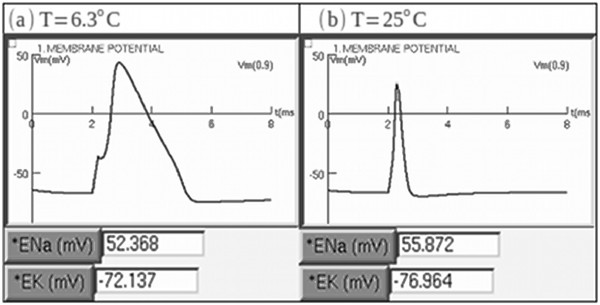
**SENB test using two different temperatures. A)** Simulation at T=6.3°C, and **(B)** simulation at T=25°C. Note that at T= 25°C, the action potential spike is narrower than at T=6.3°C owing to the increased temperature and the subsequent increase in the opening and closing rates of the sodium and potassium channels. Furthermore, |*ENa*| and |*EK*| are higher in the simulation at T=25°C than at T = 6.3°C [19].

**Figure 3:**
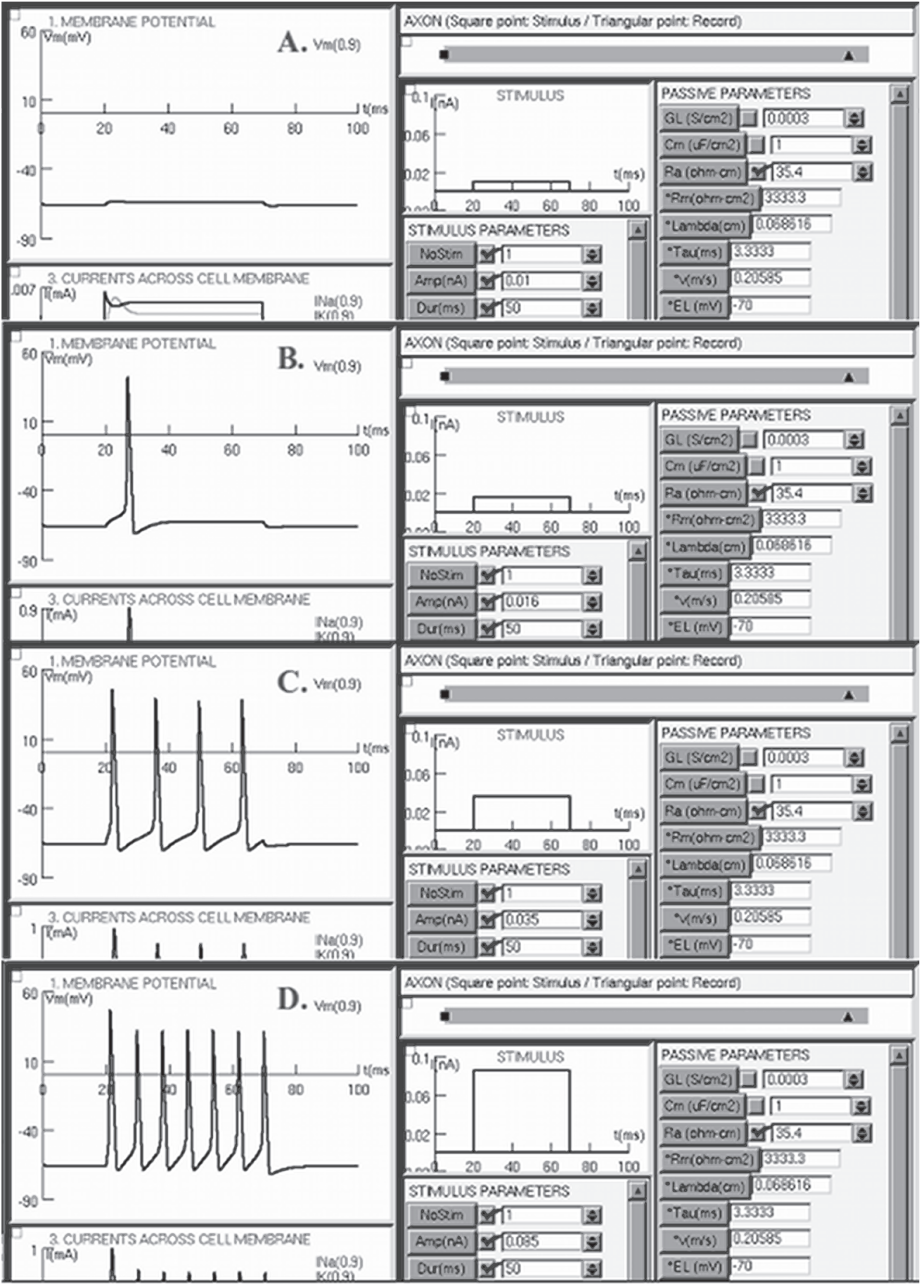
**Repetitive spiking.** Test of repetitive spiking when a long-lasting current step of constant amplitude is injected into the axon. Case **A**, graph A, small amplitude of the stimulus; case **B**, graph B, just enough amplitude to exceed the spike threshold; cases **C** and **D**, graphs C and D, amplitude is greater than the threshold.

**Figure 4:**
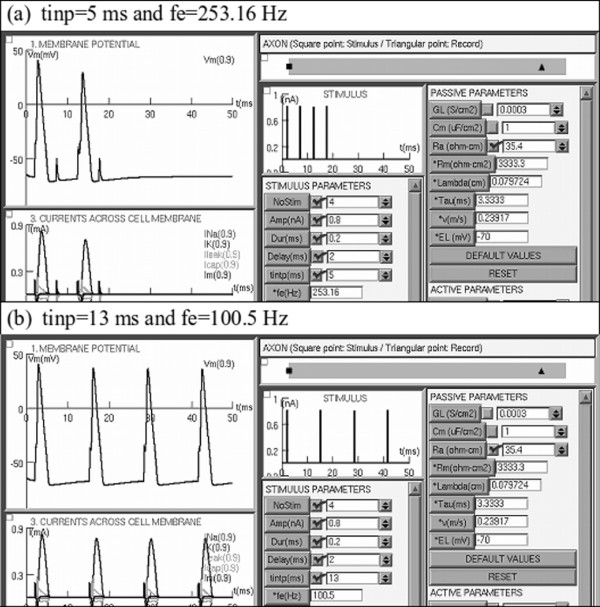
**Test of SENB using two different tintp values and then two different stimulus frequencies.** (**a**) Applying a train of four stimuli with tintp = 5 ms and fe = 253.16 Hz, and (**b**) applying a train of 4 stimuli with tintp= 13 ms and fe = 100.5 Hz. Note that at fe = 253.16 Hz, only two action potentials are propagated, while for a lower stimulus frequency, fe = 100.5 Hz, four action potentials are propagated.

The SENB software was tested using examples from scientific literature. The first example compared the results for two different temperatures, T = 6.3°C (Figure 2a) and T = 25°C (Figure 2b). The second example compared repetitive spiking when four different long-lasting current steps of constant amplitude were injected into the axon (Figure 3), while the third example compared the results for two different stimulus frequencies, fe = 253.16 Hz (Figure 4a) and fe = 100.5 Hz (Figure 4b). 

*FIRST EXAMPLE*: As T increases, the action potential spike becomes narrower and decreases in amplitude (Figure 2). This is to be expected, owing to the fact that the opening and closing rates of the potassium and sodium channels increase as T increases [19]. Then, both the time intervals for the sodium ions to enter the axolemma through voltage-dependent sodium channels and for the potassium ions to proceed to the extracellular environment through voltage-dependent potassium channels decrease. Thus, the curves of the gating variables and current densities are narrower.

*SECOND EXAMPLE*: A Hodgkin-Huxley axon stimulated with a long lasting current step of constant amplitude generates a response depending on the amplitude [25]. For a small amplitude, the response is a persistent sub-threshold depolarization (Figure 3A). If the current has just enough amplitude to exceed the spike threshold, a single action potential is generated (Figure 3B). When the amplitude is greater than the threshold, a train of spikes is generated, and the number of spikes in the train increases with the amplitude of the current (Figures 3C and 3D).

*THIRD EXAMPLE*: For a stimuli train with short length stimuli (Dur) and tintp greater than the refractory period, each stimulus generates an action potential. However, if tintp is less than the refractory period, each stimulus does not generate an action potential (Figure 4) [10,26,27]. To illustrate this phenomenon, Figure 4b shows the effect of applying a train of four stimuli with tintp = 13 ms and fe = 100.5 Hz, causing the propagation of four action potentials. Furthermore, Figure 4a shows the effect of applying a train of four stimuli with tintp = 5 ms and fe = 253.16 Hz, which causes the propagation of two action potentials.

### SENB in the computer classroom

A virtual experiment was carried out to verify the role of SENB as a facilitator for learning processes related to the spread of nerve impulses along an axon. In this experiment, a group of 33 students were asked to observe how the temperature and passive and active conduction properties affect the formation and propagation of nervous impulses. A test revealing the level of understanding of concepts of electrophysiology was given to the students before and after the virtual experiment. A paired-sample t-test, executed using the IBM SPSS Statistic 20 software, yielded the following results: p−value<0,001 (${\bar{X}}_{\text{before}}=2.82$, *σ*_*before*_=1.13; ${\bar{X}}_{\text{after}}=4.63$; *σ*_*after*_=0.53, with the grades in the range 0.00 to 5.00). Thus, the result of the test is statistically significant. The improvement in the results shows that SENB facilitates teaching and learning of the basic concepts of neuronal electrophysiology.

### Why use SENB instead of the native NEURON GUI?

There are several reasons for using SENB instead of the native NEURON GUI in the teaching and learning of electrophysiology. First, the versatile current stimuli are easily modifiable. Second, the equilibrium potentials are calculated dynamically using the Nernst equation. Third, the electrophysiological variables can be visualized in the same window, and finally, several parameters of particular importance in electrophysiology analysis are calculated by default.

#### The versatile current stimuli are easily modifiable

SENB offers greater ease and flexibility in determining the number of stimuli (current clamp). It is possible to change the number of square pulses in the current stimuli and their frequencies with just one click. The same process is applied to capture the main window as evidence.

#### The equilibrium potentials are calculated dynamically using the Nernst equation

In the native NEURON GUI, ENa and EK are independent constants of the intra- and extracellular concentrations of the sodium (nai and nao) and potassium (ki and ko) ions, respectively. In contrast, SENB calculates these parameters using the Nernst equation (Eqs. 6 and 7).

#### The electrophysiological variables can be observed in the same window

The graphical interface of SENB allows the immediate and simultaneous visualization, with the same temporal scales, of the temporal evolution of the following variables: Vm, Im, INa, IK, Icap, and the gating variables. In contrast, the graphical interface of NEURON requires that multiple windows be opened, which complicates the overall observation of results, and makes the learning and analysis of the concepts more difficult. The same problem occurs when using the graphical interface of the software “NEURON in Action”.

#### Parameters of particular importance in the electrophysiology analysis are calculated by default

Unlike NEURON, SENB by default calculates parameters such as fe, Ac, Vc, Rm, Lambda and v, the understanding of which is important to learn the electrophysiology concepts of an axon with HH properties correctly.

All of the above show the effectiveness of SENB as a didactic simulator. SENB allows not only the biophysical properties, but also the geometrical properties of an axon to be changed. This can be done in the same window (Figures 1 and 3), which is divided into several sections to facilitate the observation and location of graphs, buttons, and boxes, and thus the interpretation of the results.

In the same vein, it is worth highlighting that SENB allows immediate visualization of the effect of variations in the conduction axonal parameters, namely, Vm, Im, INa, IK, Ileak, Icap, m, n, h, m^3^, m^3^h, and n^4^. This facilitates the students’ analysis, comprehension of the basic concepts of neuronal electrophysiology, and interaction with the electrophysiological variables.

The educational benefits that are derived from using SENB could be improved if a set of virtual experiments were designed according to the requirements and scope of a specific course. Furthermore, other strategies could be applied, depending on the creativity and needs of each class, to guide the students’ work and encourage active participation in the learning process of electrophysiology. These strategies should guide the students through the understanding of not only the basic concepts related to neuronal electrical conduction, but also the equations describing the action potential along the axon.

## Conclusions

SENB is a didactic versatile computational tool developed using NEURON’s interpreted programming language *hoc*. It can be used to improve and facilitate the teaching and learning of the basic concepts of neuronal electrophysiology.

SENB simulates the propagation of action potentials along a model of an unmyelinated axon with electrical conduction properties like the squid giant axon. Using a single window as the graphical interface, the axonal biophysical and geometrical properties can be changed. Furthermore, the software allows for the immediate and simultaneous visualization with the same temporal scales of the evolution of the current densities, membrane potentials, and gating variables.

SENB by default calculates parameters such as the frequency of stimuli, propagation velocity of action potentials, space and time constants, axonal area and volume, and equilibrium potentials.

Generally, SENB is an excellent alternative for teaching and learning processes related to the underlying mechanisms in the electrical activity of an axon with HH properties. It offers students multiple options for changing the electrical neuronal properties and immediate observation of the effects thereof.

## Availability and requirements

**Project name:** Software development in NEURON simulation environment for the teaching of electrophysiology**Operating system(s):** Linux**Programming language:** NEURON’s interpreted programming language *hoc***Other requirements:** NEURON**Any restrictions on the use by non-academics:** No license is required, only the author’ permission

## Competing interests

Both authors declare that they have no competing interests.

## Authors’ contributions

OH and EZ wrote, read, and approved the final manuscript. OH developed the software. OH and EZ executed the software and analyzed the results and educational impacts. Both authors read and approved the final manuscript.

## Authors’ information

Oscar E. Hernández received a BS degree in Mathematics and Physics from Universidad del Atlántico (Barranquilla, Colombia) in 2001, an MS degree in Medical Biophysics from the Universidad de Chile, and is currently a student in the Ph.D. program of Industrial Engineering at Universidad del Norte. Since 2005 he has been a Professor in the Department of Chemistry and Biology (Departamento de Química y Biología) at the Universidad del Norte.

Eduardo E. Zurek received a BS degree in Systems Engineering from the Universidad del Norte (Barranquilla, Colombia) in 1994, an MS degree in Electrical Engineering from the University of South Florida in 2002, and a Ph.D. degree in Electrical Engineering from the University of South Florida in 2006. Since 1994 he has been a Professor in the Department of Systems Engineering (Departamento de Ingeniería de Sistemas) at the Universidad del Norte.

## Pre-publication history

The pre-publication history for this paper can be accessed here:

http://www.biomedcentral.com/1472-6920/13/70/prepub
